# DNA read count calibration for single-molecule, long-read sequencing

**DOI:** 10.1038/s41598-022-21606-5

**Published:** 2022-11-01

**Authors:** Luis M. M. Soares, Terrence Hanscom, Donald E. Selby, Samuel Adjei, Wei Wang, Dariusz Przybylski, John F. Thompson

**Affiliations:** Genomics and Computational Biology, Homology Medicines Inc, Bedford, MA USA

**Keywords:** Genetics, Sequencing, DNA sequencing, Next-generation sequencing

## Abstract

There are many applications in which quantitative information about DNA mixtures with different molecular lengths is important. Gene therapy vectors are much longer than can be sequenced individually via short-read NGS. However, vector preparations may contain smaller DNAs that behave differently during sequencing. We have used two library preparations each for Pacific Biosystems (PacBio) and Oxford Nanopore Technologies NGS to determine their suitability for quantitative assessment of varying sized DNAs. Equimolar length standards were generated from *E. coli* genomic DNA. Both PacBio library preparations provided a consistent length dependence though with a complex pattern. This method is sufficiently sensitive that differences in genomic copy number between DNA from *E. coli* grown in exponential and stationary phase conditions could be detected. The transposase-based Oxford Nanopore library preparation provided a predictable length dependence, but the random sequence starts caused the loss of original length information. The ligation-based approach retained length information but read frequency was more variable. Modeling of *E. coli* versus lambda read frequency via cubic spline smoothing showed that the shorter genome could be used as a suitable internal spike-in for DNAs in the 200 bp to 10 kb range, allowing meaningful QC to be carried out with AAV preparations.

## Introduction

Long-read sequencers have been a boon for genomic studies by allowing long-distance sequence connections to be obtained directly and by providing single-molecule resolution of DNA and RNA species^1,2^. These technical advances have enabled genome assembly, structural variation and mRNA isoforms to be more rapidly and precisely understood^2,3^. For the most part, the focus of long-read sequencing technology has been on producing the longest reads possible, allowing easier genomic assembly and structural insight^4–6^. When using these technologies, shorter reads are often considered a nuisance that interfere with maximizing the number of long reads and methods have been adapted to avoid them to the extent possible. Base-calling accuracy has been important, but generally not the critical feature in such systems.

When trying to assemble genomes, characterize structural variation, or examine long cDNAs, the read length maximization goal is reasonable. However, there are other sequencing applications such as RNA-seq, Chip-seq, and analysis of short genomes where reads beyond a particular length threshold are not useful, so length maximization loses significance and other factors become more important. A sequence longer than the hundreds of bases generated by short-read NGS technologies may be required but reads in the hundreds of thousands of bases are not necessarily useful.

One application where only modest read lengths are necessary is the characterization of Adeno-associated Virus (AAV) DNA samples. Full-length, wild-type AAV is ~ 4.7 kb long and recombinant versions (rAAV) are frequently used as gene therapy vectors^7,8^. Knowledge of their purity and sequence is of critical importance prior to use in clinical situations^9,10^. Knowing which DNA contaminants could be present and their amounts requires having accurate quantitative information across the size range of hundreds to thousands of base pairs. DNA contaminants could arise from many sources including from rearrangements of the desired vector, from packaging cell line genomic DNA, or from plasmids used for generating the vector. Sequence information provides the data necessary for identifying the source of the DNA, but it is also important to characterize the stoichiometry of those DNAs. The potential contaminants could have a wide range of sizes so knowing how all DNAs behave in library processing and NGS is important for quantitatively assessing the composition of any preparation. Similar issues can be envisioned with other DNA vectors as well as with other applications. Incomplete knowledge about how efficiently different DNA molecules are sequenced could lead to erroneous DNA frequency estimations.

Both Pacific Biosciences (PacBio) and Oxford Nanopore NGS easily provide read lengths that meet the needs of AAV sequencing so either can be used for assessing AAV samples. Both technologies have been successfully employed for sequencing AAV with a variety of library preparation methods^11–14^. Tai et al.^12^ calibrated the length dependence of their library preparation and sequencing methods using lambda DNA. However, lambda is only 48 kb so may not provide a large enough number of DNA fragments for a high-resolution length map. To address this issue, we have used a much longer DNA, *E. coli*, as a calibration marker to generate hundreds of DNAs that should be present at approximately equimolar ratios. These DNAs have been examined via standard library preparation methods with both PacBio and Oxford Nanopore NGS systems to determine how to best quantitate rAAV and its potential contaminants. We have aimed our analysis at the DNA size range relevant to AAV and its potential contaminants, 200 bp to 10 kb. The same methods could be used for applications with other DNAs of varying sizes with similar needs.

## Materials and methods

### Reagents

All restriction enzymes, heat-labile Protease K and lambda DNA were purchased from New England Biolabs. When using different restriction enzymes for samples and spikes, care must be taken to avoid cross-digestion of DNAs. Some enzymes, like PvuII, are not readily heat-killed and may persist even after bead clean-up. Treating the sample with heat-labile Protease K and then heat-killing the protease is advisable in such situations. Standard library generation and sequencing kits and protocols were purchased from PacBio (Template Prep Kit: SMRTbell Express Template Prep Kit 2.0; Binding Kit: Sequel II Binding Kit 2.0 or Sequel II Binding kit 2.1; Sequencing Kit: Sequel II Sequencing Plate 2.0; DNA Control: Sequel II DNA Internal Control 1.0; PacBio SMRTcell Version: SMRT Cell 8 M (REF: 101–389-001)) and Oxford Nanopore (SQK-RAD004 for transposase libraries and SQK-LSK109 for ligation libraries; ONT Flowcell Type: Spot-ON Flow Cell, R9 Version (FLO-MIN106D)) and used as directed. PacBio samples were barcoded using Barcoded Overhang Adapter Kit 8A (REF: 101–628-400) and 8B (101–628-500) and sequenced for 30 h on a Sequel II. Oxford Nanopore samples were barcoded using ONT Barcode Kit: Native Barcoding Expansion 1–12 (EXP-NBD104) and Native Barcoding Expansion 13–24 (EXP-NBD114) and sequenced for 72 h on MinIons. Relative sizing for DNA mixes was determined using an Agilent 5300 96-well Fragment Analyzer capillary electrophoresis instrument using the DNF-468 HS Genomic DNA 50 kb kit with a GQN cutoff of 10,000 bp.

### Biological resources

*E. coli* K-12 MG1655 genomic DNA and cells were purchased from ATCC. To generate stationary phase DNA, *E. coli* K-12 MG1655 cells were grown overnight in 250 mL Terrific Broth. Cells (OD600 = 4.5) were spun down for genomic DNA preparation. For exponential phase DNA, a 10 μL aliquot of the stationary phase cells was diluted into 250 mL Terrific Broth and grown for ~ 4 h at 37C and exponential phase *E. coli* cells (OD600 = 0.214) spun down. *E. coli* genomic DNA was prepared using the Zymo Research Quick DNA HMW MagBead kit.

### Data availability

Sequence data was acquired using a PacBio Sequel II and Oxford Nanopore software. Data has been deposited at SRA with accession numbers SAMN28890519, SAMN28890520, SAMN28890521, SAMN28890522, SAMN28890523, SAMN28890524, SAMN28890525, SAMN28890526, SAMN28890527, SAMN28890528, SAMN28890529, SAMN28890530, SAMN28890531, SAMN28890532, SAMN28890533, and SAMN28890534.

### Sequence analysis-PacBio

All analysis of PacBio data was performed on Consensus Circular Sequencing (CCS) reads. CCS calling was performed on the original subread bam files from PacBio Sequel II instrument using SMRT-Link version 9.0 CCS tool with the following parameters:–minLength 10–maxLength 50,000–minPasses 3–minSnr 2.5–maxPoaCoverage 0–minPredictedAccuracy 0.99, except when comparing 1 vs 3 full passes in which case the–minPasses parameter was adjusted accordingly and the minimum predicted accuracy was set to 0. Demultiplexing of samples was performed with the lima tool from the same version of SMRT-Link using the ccs preset and a min-score parameter set to 0. References for alignment were created using *E. coli* MG1655 (NC_000913) genome and *E. coli* phage Lambda (J02459) genome digested with the corresponding restriction enzyme so that each fragment is an entry in the reference file. Alignment to references was performed using pbmm2 align mode with CCS preset. Reads that produced supplementary alignments (usually caused by incomplete digestion) were removed from downstream analysis. Some reads with CCS = 1 are not considered high quality and have variable qualities, but we find that the size of the reads produced by the PacBio instrument overcomes any issues with mappability related to base qualities. The issue of mappability, if it were to occur, would be more pronounced in short reads (which are excluded by the library preparation methods). In addition, the *E. coli* genome used as reference for these experiments has limited complexity and issues with mappability are limited to a very few digestion fragments that create non-unique fragments. Bioinformatics analysis was performed using custom python scripts that can be found at https://bitbucket.org/HMI_BB/long_read_calibration/src/master/ (packages: pysam (0.17.0), numpy (1.21.2), pandas (1.2.4), sklearn (1.0.2), scipy (1.7.1) and csaps (0.11.0)). Bioinformatics analysis include dataset statistical description and splitting, read filtering and properties quantification (size, quality, corresponding reference), reference coverage calculations, regression analysis and visualization.

### Sequence analysis—Oxford nanopore

Oxford Nanopore raw data was base called using Guppy version 5.0.11 in accurate mode (dna_r9.4.1_450bps_hac.cfg) and demultiplexed using guppy_barcoder with–trim_barcodes option. Adapters were trimmed using porechop with–discard_middle option. Alignment of reads to reference genomes was performed using mini_align (minimap 2.17-r941 wrapper) with the -A flag set. Reads that produced supplementary alignments were removed from downstream analysis and only remaining primary alignments were considered. Bioinformatics analysis was performed using custom python scripts that can be found at https://bitbucket.org/HMI_BB/long_read_calibration/src/master/.

### Algorithms (regression analysis)

For the linear regression analysis, functions from the scikit-learn Python toolkit were used. For the cubic spline smoothing, CSAPS (https://github.com/espdev/csaps) was used. This is an implementation of the MATLAB CSAPS function that was inspired by the Fortran routine SMOOTH from PGS (originally written by Carl de Boor).

## Results

To generate significantly more DNA markers than possible with the 48,502 bp lambda bacteriophage DNA, the 4,641,652 bp *E. coli* genomic DNA (NC_000913) was used to create ~ 100 × more equimolar markers of varying sizes. Restriction enzymes were chosen to generate DNA of defined size in the range most relevant to rAAV studies. To include the potential impact of varying end sequences that would be found with real DNA samples, some enzymes were chosen with cleavage sites having degenerate cleavage sequences so that all possible terminal variations could be tested. In addition, PvuII was used so that a set of fragments with the same terminal bases could be examined. Enzymes used are shown in Table 1. The predicted cuts differ from the unique cuts due to identical fragments being generated within the ribosomal repeat regions. In addition, not all cuts are feasible when multiple recognition sequences occur within a very short genomic region. *E. coli* DNA from the sequenced strain MG1655 was cut separately with AleI, PshAI, PvuII and XmnI. Because of the large number of products, digest completion could not be assessed by standard electrophoresis-based methods, but it was confirmed via sequence information. About 1% of fragments were not completely cut with PshAI, PvuII and XmnI, while approximately 7% of AleI fragments were incompletely cut. The issue with AleI is caused by its recognition site overlapping the EcoKI methylation recognition site (AAC(N)_6_GTGC), an active methylase in this strain of *E. coli.* All AleI sites include at least four base pairs in common with EcoKI and sometimes include all seven bases. The size of fragments containing the complete EcoK1 recognition sequence indicate that methylation prevents AleI cutting. A small number of other AleI sites are also resistant to cleavage.

**Table 1:** Enzymes used for generating standards.

Enzyme	Recognition Site	Predicted *E. coli* Cuts	Unique	Size Range (bp)
Ale I	CACNN ^ NNGTG	888	880	2– 32,955
PshAI	GACNN ^ NNGTC	676	675	4– 43,780
XmnI	GAANN ^ NNTTC	1719	1715	11– 19,101
PvuII	CAG ^ CTG	1776	1776	6– 21,759

Two different standard library preparation methods were used for both PacBio and Oxford Nanopore. Protocols 2.0 (longer DNA reads) and 2.1 (shorter DNA reads) were used for PacBio. Both these methods include ligation of a SMRTbell hairpin to the ends of each molecule to be sequenced. The primary difference between the methods is the DNA purification step in which the ratio of SPRI beads used is more (2.1) or less (2.0) favorable for retaining short DNA fragments. The differences between the Oxford Nanopore methods are more significant. The ligation-based method attaches sequencing adaptors to the end of each molecule like the PacBio methods while the rapid method uses a transposase to insert sequencing adaptors into random internal locations within the DNA. The ligation method has the potential to generate reads matching the full-length DNA while the transposase method will necessarily shorten the DNA on one end due to the internal insertion so individual reads will not directly provide the original molecular length.

The relative sizes of some DNA samples that were restriction cut and purified via PacBio Protocol 2.0 or 2.1 were evaluated on a Fragment Analyzer to determine the degree of preferential removal of the smaller fragments. The large number of bands prevents direct comparison of individual DNA fragments, so the Genome Quality Number (GQN) score calculated by the instrument was used as an approximation of relative DNA size with larger GQN scores indicating larger DNA. *E. coli* genomic DNA purchased from ATCC and DNA purified from exponential phase cells was of similar size while DNA from stationary phase cells was smaller, possibly due to degraded DNA from dead cells (Table 2). Pooled, digested DNA purified using protocol 2.0 yielded GQN = 1.1 and DNA purified using protocol 2.1 yielded GQN = 0.7, confirming that the 2.1 prep retains shorter DNAs that the 2.0 prep removes. Fragment Analyzer traces for the DNA after purification but prior to SMRTbell attachment are shown in Supplemental Fig. 1 where DNA towards the right is larger and includes a greater fraction of all DNA in Protocol 2.0 relative to Protocol 2.1. When the starting lambda DNA sample is examined, nearly all DNA appears to be larger than 48 kb. Because of the sticky ends at the lambda cosN sites, untreated lambda tends to form long concatemers when full length molecules hybridize to each other. To prevent this, we filled in the sticky ends with Taq polymerase. The same amount of DNA was run on the Fragment analyzer (Supplemental Fig. 2) but the filled in DNA appears shorter though still longer than 48 kb. Most of the untreated DNA is not detected in the Fragment Analyzer because it is too large. This indicates the lambda DNA we are using is predominantly full length. The uncut lambda DNA has a lower GQN value because the shorter random fragments play a larger role in the calculation.

**Table 2:** Fragment Analyzer Relative Lengths with GQN10 cutoff.

Treatment	DNA	Source	GQN/Replicate
None	*E. coli*	ATCC	9.2/8.7
None	*E. coli*	Stationary	6.8/6.8
None	*E. coli*	Exponential	9.1/9.2
XmnI	*E. coli*	Stationary	0.7
XmnI	*E. coli*	Exponential	0.9
PvuII	*E. coli*	Stationary	0.8
PvuII	*E. coli*	Exponential	1.4
XmnI	Lambda	NEB	0.2
Lib 2.0	*E. coli,* Lambda	Pooled	1.1
Lib 2.1	*E. coli,* Lambda	Pooled	0.7
Uncut	Lambda	NEB	8.7
Uncut, filled in	Lambda	NEB	9.7

**Figure 1:**
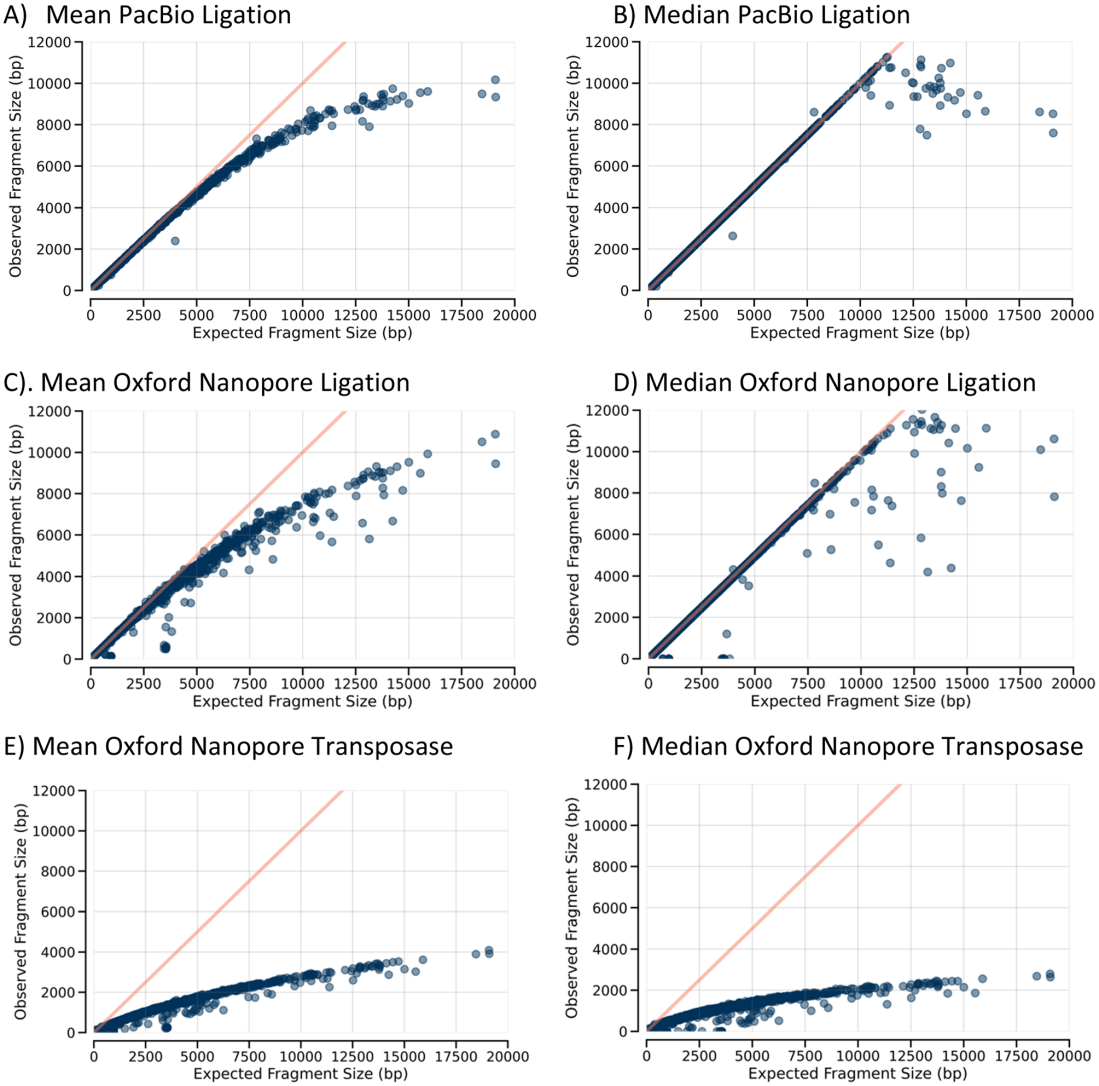
Observed sizes versus expected sizes for XmnI digests. Expected versus observed lengths of XmnI-cut *E. coli* stationary phase DNA. The expected length of XmnI fragments prepared using PacBio library method 2.1 derived from the *E. coli* genome sequence is plotted on the X-axis versus the mean (**A**) or median (**B**) size of observed fragments from stationary phase DNA. The mean and median for the same DNA prepared using the Oxford Nanopore ligation method (**C** and** D**) and the transposase method (**E** and** F**) are also shown.

**Figure 2:**
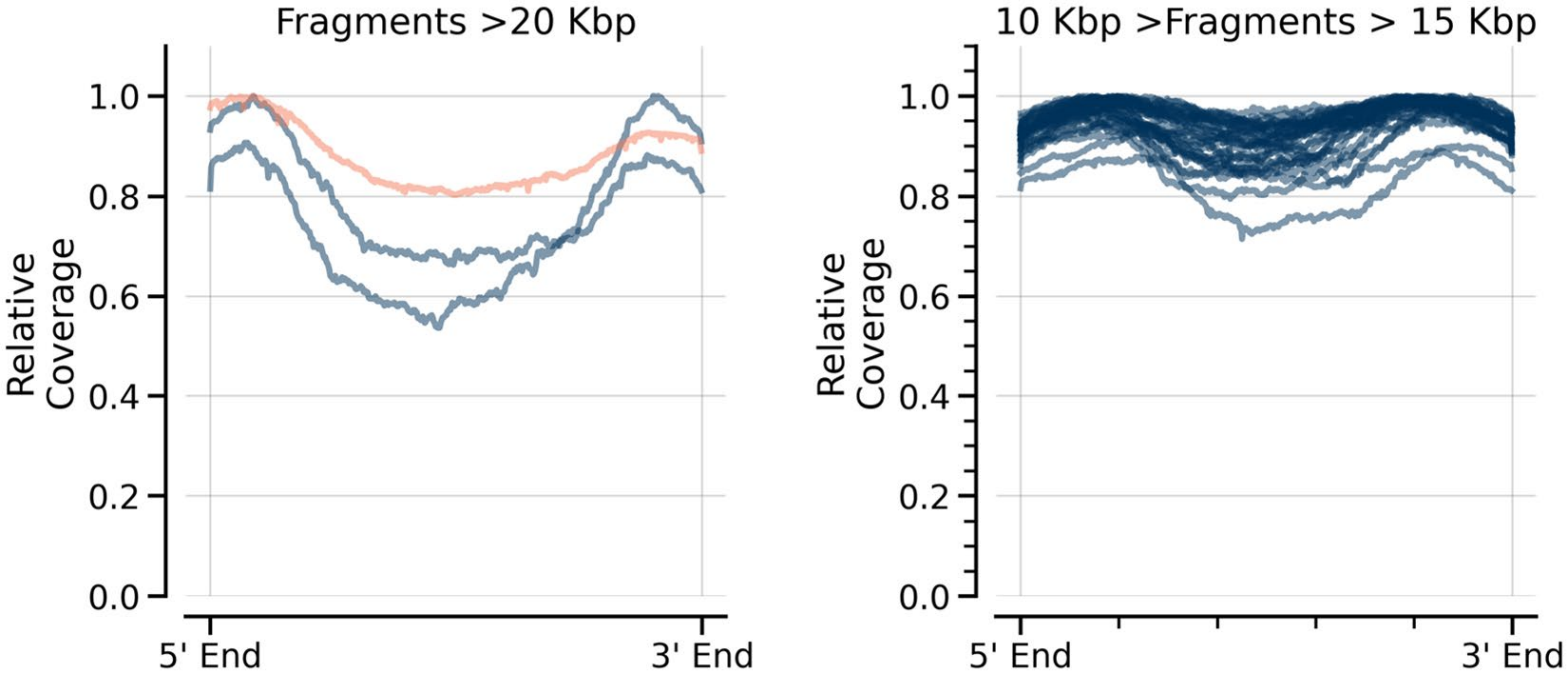
Coverage of long fragments. The relative coverage of individual DNA fragments is shown for *E. coli* (blue) and lambda (orange). Each line represents an individual PvuII fragment from either *E. coli* (blue) or lambda (orange). With PvuII, there were no lambda fragments with a size of 10–15 kb while the 30 such *E. coli* fragments are each shown individually in blue. Coverage across the length of each fragment is normalized to the maximum for the given DNA and then plotted versus the normalized length. The dip in coverage in the middle of the fragments is most extreme for long *E. coli* fragments.

Figure 1 shows the mean (Fig. 1A) and median (Fig. 1B) fragment lengths of reads generated using PacBio 2.1 for XmnI restriction fragments relative to the expected lengths of the fragments they map to. Up to about 4,000 bp, the mean fragment lengths and corresponding reference match precisely. With longer DNAs, the fragment length increasingly deviates from the expected length, reaching a maximum observed length of ~ 10,000 bp independent of the expected length of the molecule. The median of observed fragment sizes is better behaved, maintaining their expected sizes up to around 10,000 bp. Taken together, these observations point to a size of around 4,000 bp at which truncated reads start to become increasing prevalent though most fragments up to 10,000 bp remain intact. This observation can be explained by random fragmentation of the DNA that occurs during purification and/or processing. No special care was taken during handling of samples to ensure maximum DNA length, so this size range is expected. However, it does indicate that DNAs predicted to be larger than 10 kb may have more than a single molecule that could be sequenced so the equimolar DNA ratios will shift from the expected value in that size range. The Oxford Nanopore ligation method (Fig. 1C and D) has very similar results relative to the PacBio though with slightly more variability. As shown in Supplemental Fig. 2, most DNAs are full length using the ligation-based method. The transposase-based method (Fig. 1E and F), however, is starkly different. Because the sequencing adaptors are inserted randomly within the fragments, the lengths are typically half that of the ligation methods.

To further address the behavior of long DNA fragments, the behavior of a long lambda DNA fragment was compared to *E. coli* DNA fragments within the same sequencing library. Despite combining DNAs prior to library preparation, coverage differences between the samples are instructive. As shown in Fig. 2 with the three PvuII fragments predicted to be > 20 kb, the coverage of the lambda fragment (21,299 bp, orange) was more even than the coverage of the two *E. coli* fragments (21,759 and 20,270 bp, blue). The thirty 10–15 kb *E. coli* fragments also had more even coverage (shown individually in Fig. 2). This is likely due to the starting lengths of the two genomes. The 48 kb lambda will be randomly sheared only rarely during sample handling while the 4.6 Mb *E. coli* will be sheared throughout its length during handling with breaks randomly occurring more often in longer fragments. This difference highlights the limitation that control DNAs may not always reflect the behavior of test DNAs, even when handled identically.

**Figure 3:**
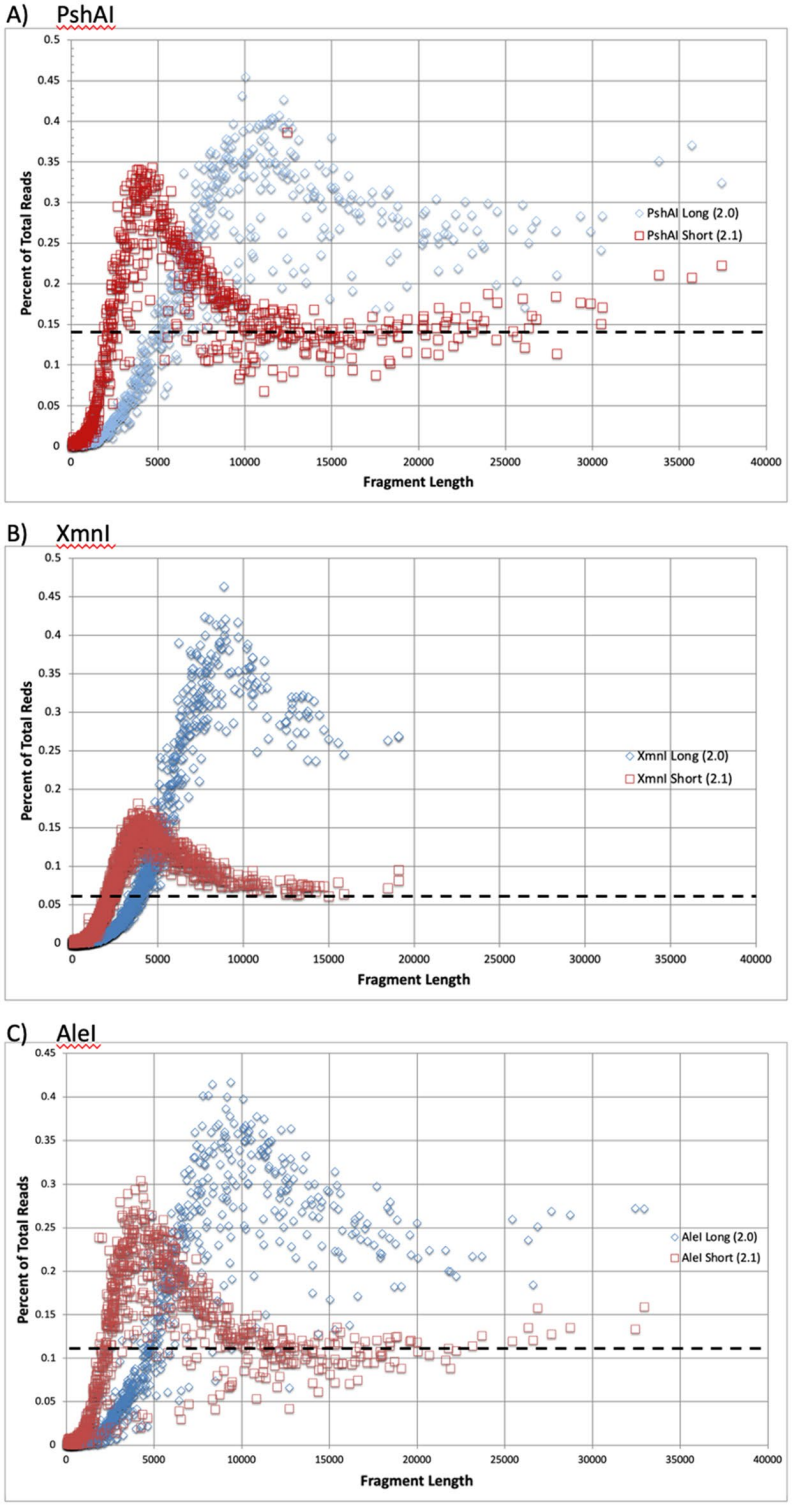
Read frequency as a function of DNA length. The number of reads for each DNA was normalized to the total number of reads for the whole library and then plotted as a function of predicted DNA length. For each enzyme, the short library (protocol 2.1) is shown with red squares and the long library (protocol 2.0) with blue circles. The expected read count for each library if fragment recovery and sequencing were perfect for all fragments is shown by the dashed line in each panel.

For the libraries summarized in Table 3, the length and frequency for each individual DNA are shown in Fig. 3 for samples cut with PshAI (Fig. 3A), XmnI (Fig. 3B) and AleI (Fig. 3C). Individual reads for each short library (protocol 2.1) are shown in red and for each longer library (protocol 2.0) in blue. The percent of total reads for each fragment in each sample is plotted versus the expected DNA size. As shown in Fig. 3, a complex behavior is evident for read frequency as a function of length. The overall shape of each library is similar with frequency rising as the DNAs get longer, reaching a peak for expected length of ~ 4000 bp for the 2.1 short preps and ~ 10,000 bp for the 2.0 long preps. For both protocols, the frequency then drops before plateauing around 15,000 bp. All libraries are normalized with the percent of reads for each fragment relative to all reads in the library. The apparent higher level of reads with the long method is caused by the absence of short DNAs whose reads are redistributed to the longer DNAs. In general, the read frequency at a given length is within a two-fold range, making it possible to predict length-dependent differences in sequencing efficiency with that limit of accuracy for each protocol. If all fragments were recovered and sequenced equally well, all fragments would fall on the dashed line. Deviations above the line show the preferential sequencing of some DNAs while deviations below the line show the reduced sequencing efficiency of shorter fragments.

**Table 3:** Relative behavior of DNA in different PacBio Libraries.

Library Prep	Unique Cuts	# Fragments with reads:			Max %	Median Length of Top 20	Size Range of Top 20 (bp)
		> 0.001%	> 0.01%	> 0.1%			
AleI Long (2.0)	880	840	626	359	0.417	9479	7771– 12,819
AleI Short (2.1)	880	850	737	512	0.304	4101	3068– 5540
PshAI Long (2.0)	675	670	511	352	0.455	11,564	9349– 43,780
PshAI Short (2.1)	675	668	601	482	0.386	4112	3435– 12,456
XmnI Long (2.0)	1715	1493	802	318	0.463	8711	6215– 10,261
XmnI Short (2.1)	1715	1661	1146	504	0.181	4315	3526– 5246

Reads from each restriction digest (AleI, PshAI, and XmnI) were compared for both PacBio library preparations, prep 2.0 which favored longer DNA molecules and prep 2.1 which also included many shorter DNA molecules. The number of unique fragments is in column 2 while the number of DNAs with aligned reads are shown in columns 3–5 with the frequency of the DNA molecule with the highest read count listed in column 6 (Max %). The median (column 7) and range (column 8) of lengths for the 20 most frequent DNAs in each library are listed to show the most effective sequencing range for each protocol.

The enzymes used have different cleavage site frequencies and the resultant DNA digests span broad size ranges. The relative frequency of the same size fragments is similar among all enzymes. This is consistent with DNA size rather than sequence being a main driver of read frequency. Selective loss of different sized DNAs by varying SPRI conditions in the 2.0 versus 2.1 library preparation methods generates a different size profile for the same starting DNAs due to selective loss of shorter DNAs. As shown in Table 3, nearly all individual DNA fragments within the sample have some (> 0.001%) reads, but the number of more frequent fragments (> 0.01% reads) drops markedly in the long (2.0) library prep samples and even more so for fragments with > 0.1% reads. The lengths and frequencies of the most commonly sequenced DNAs are always higher in the long preps while the short preps retain a broader selection of DNAs. The maximum read percent is always lower with the short library because the reads are distributed over a larger number of DNAs.

**Figure 4:**
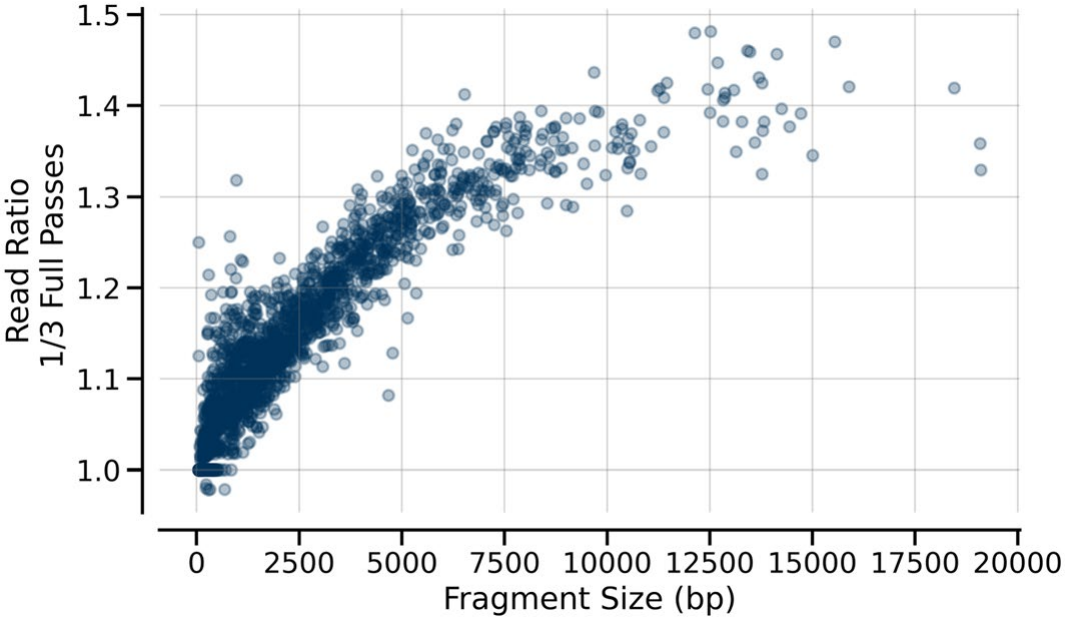
Impact of DNA size on CCS read frequency. The ratio of reads with CCS cycle set to 1 versus 3 is shown as a function of DNA size. At sizes of 1000 bp and below, there is little or no effect. The impact grows progressively larger with longer DNA sizes.

Another factor that can affect the apparent yield of different length DNAs using PacBio NGS is the mode of sequencing. If Circular Consensus Sequencing (CCS) is used, a set number of cycles of sequencing around the circularized DNA is used to improve the sequence accuracy (for more detail on the CCS method, see https://www.pacb.com/videos/tutorial-circular-consensus-sequence-analysis-application/). However, this choice can have a substantial impact on the number of longer reads as multiple cycles will be less likely to be able to complete within any given sequencing time period. Molecules that are not able to complete the requisite number of sequencing cycles are not counted so fewer cycles allow counting of more molecules. The effect of varying the number of CCS cycles should affect all DNAs of the same size in the same way so DNAs used in the calibration will mirror the impact on the test DNAs. Setting CCS = 1 provides both more reads and less variable counting though at the expense of base-calling accuracy. As shown in Fig. 4, changing from CCS = 3 to CCS = 1, had up to a 1.5-fold effect on read count with this set of conditions and the effect is more substantial with longer DNAs than with short DNAs.

The concept of a perfectly equimolar ratio of DNA fragments requires complete cutting of the targeted genome, but it also requires that the genome itself is present at a fixed integer value. This is more readily achieved with viral DNAs like lambda, but it can be more challenging for longer genomes. As shown in Supplemental Fig. 3, the fragment analyzer trace for lambda shows no significant shorter fragments. In fact, it is considerably larger than the expected 48,502 bp length because the 12 nt sticky overhangs at the cosN site cause concatemerization of multiple full-length molecules. Concatemerization is only possible if both cosN sites are present. When intermolecular hybridization is prevented by filling in those ends with Taq polymerase, the DNA appears shorter but still full-length (Fig. S3). Thus, there may be a small amount of fragmented or recombined DNA, but this lambda DNA can be approximated as being uniformly full-length. For non-growing bacterial cells like stationary phase *E. coli*, there is likely a single, complete copy of the genome. For a rapidly growing bacterial cell, the rate of genome replication may affect copy number. For example, *E. coli* has a single origin of replication region that is replicated prior to the rest of the genome. Sequences near the origin of replication (3,925,744–3,925,975)^15^ will be over-represented relative to more distal parts of the genome. To assess this, DNA fragments from multiple restriction digests were used to determine whether growth phase affected stoichiometry in a genomic position-dependent manner.

**Figure 5:**
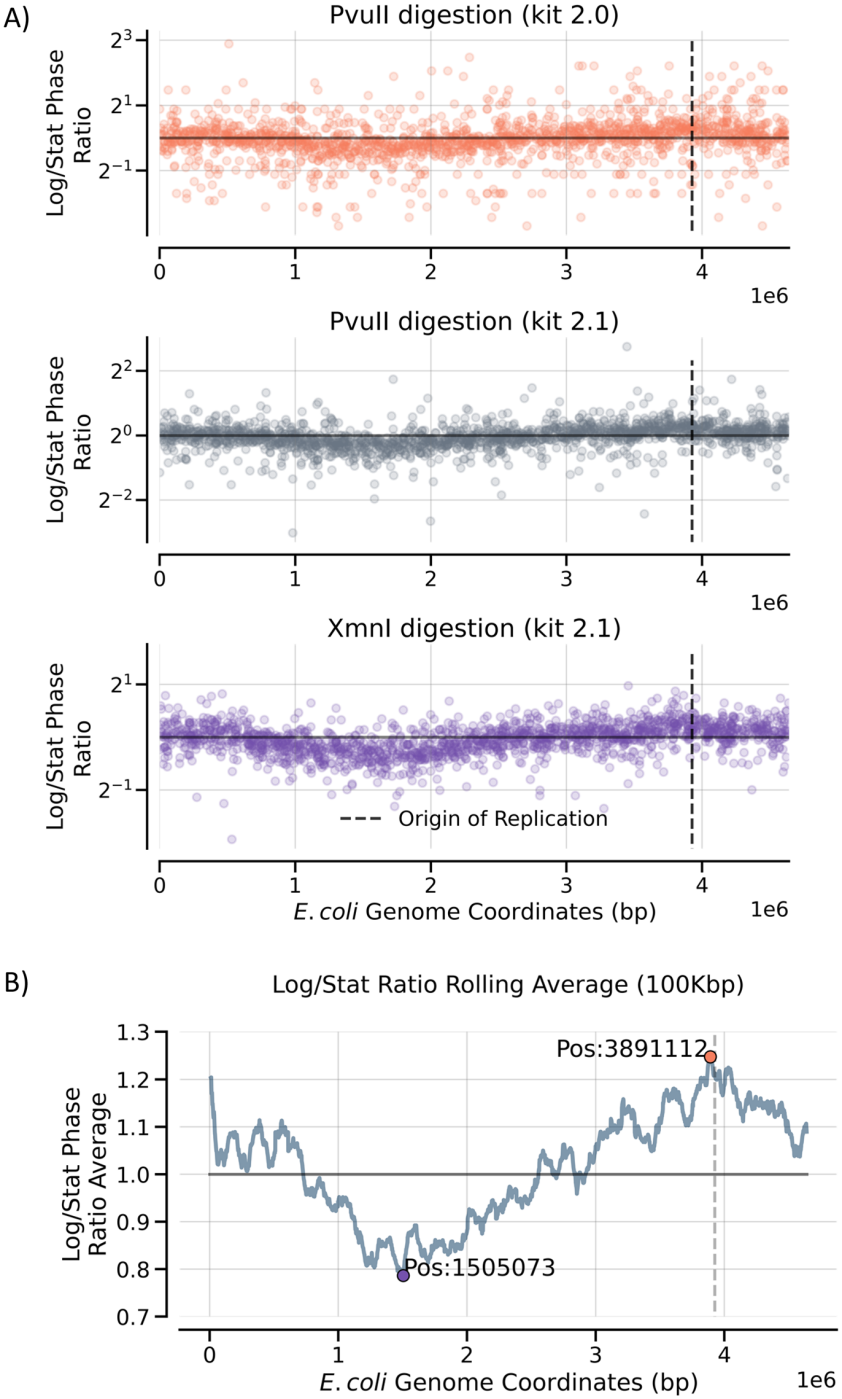
Ratio of normalized reads in exponential vs. stationary phase *E. coli* genomes. Reads from three digests were normalized within the exponential and stationary phase DNA samples. The ratio of exponential/stationary phase reads for each fragment as a function of genomic position is shown in panel (**A**). The results from all three digests were then combined and the ratios averaged over bins of 100,000 bp as shown in panel (**B**).

*E. coli* was grown overnight to a saturated, stationary phase and some cells used for DNA extraction. In addition, an aliquot of the stationary phase cells was diluted, grown exponentially, and the DNA extracted from exponential phase cells. Reads were normalized within each sample and the ratio of exponential/stationary phase reads for each fragment determined. Those ratios are plotted as a function of genomic location, either individually (Fig. 5A) or as a rolling average of ratios within 100,000 bp bins with all three digests combined (Fig. 5B). This approach smooths the variation arising from individual fragments. Consistent with the known origin and termination of replication locations, there are relatively more DNA reads centered around the 3.9 Mb position and correspondingly fewer on the other side of the genome near 1.6 Mb. A very similar pattern is generated when a weighted, rolling average of adjacent fragments is used rather than bins of defined lengths (Supplemental Fig. 4).

With the rolling bin size average of combined samples, the position with the maximum ratio of exponential to stationary phase reads occurs at position 3,891,112 while the position with the minimum ratio occurs at 1,505,073 (data for individual digests in Table 4). The maximum to minimum frequency ratio of exponential/stationary phase reads is 1.68. When the reads near the origin or near the termination sites (either fixed number of DNAs or fixed distance) are compared for stationary versus exponential phase, the differences are highly significant ((*p* < 0.00001). The same is true for the ratio of reads near the origin versus termination sites for exponential DNA. However, the stationary phase origin reads relative to the termination site reads are only marginally significant (*p* ~ 0.05). This reflects the much more even genomic coverage observed with stationary phase DNA relative to exponential phase DNA. Thus, if *E. coli* is to be used as an equimolar control, use of stationary phase DNA is preferable from a stoichiometric perspective but less so from a genomic length/shearing point of view. At worst, growth phase will have only a modest effect on results.

**Table 4:** Ratios of Exponential to Stationary Phase Reads.

	PvuII (2.0)	PvuII (2.1)	XmnI (2.1)
Minimum Ratio	0.749	0.750	0.794
Genomic Position (min)	1,674,917	1,677,699	1,887,059
Maximum Ratio	1.245	1.218	1.282
Genomic Position (max)	4,046,211	3,945,465	3,891,112
Ratio Max/Min	1.661	1.625	1.615

**Figure 6:**
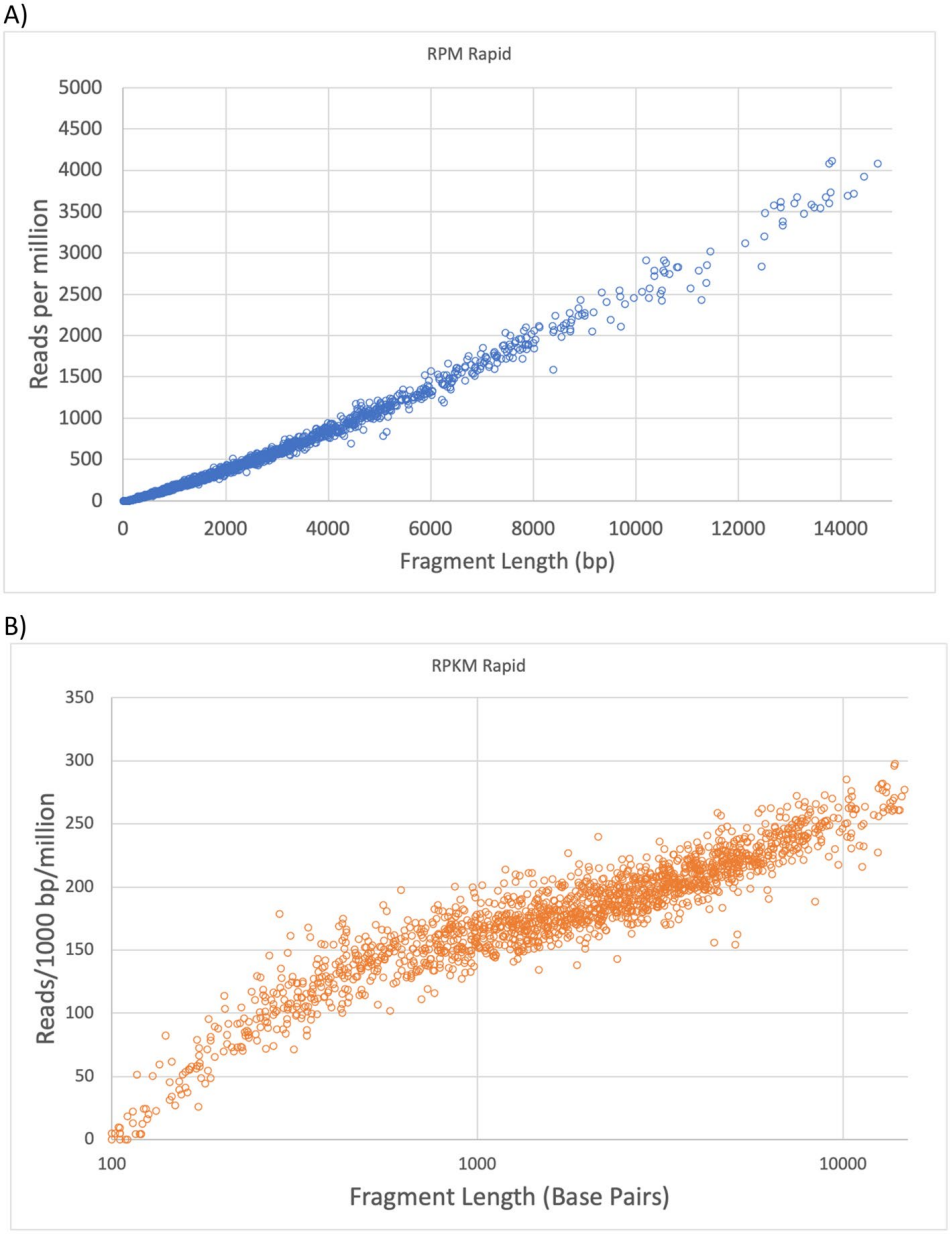
Oxford Nanopore reads using the transposon-based fast library preparation. XmnI-cut *E. coli* DNA was prepared for sequencing using the standard fast library preparation method. The number of RPM is plotted as a function of predicted DNA length (**A**). The same data set was converted to RKPM to adjust for length and plotted again (**B**) but on a log scale.

Oxford Nanopore utilizes a completely different technology for generating long reads and they provide two distinctly different library preparation methods. One method employs a transposase to attach sequencing adaptors to relatively random positions within the target DNA. This approach should generate read numbers in proportion to the length of the DNA because, in theory, every DNA position has an equal chance of insertion unlike ligation where there are always two ends, independent of length. This is the case as shown in Fig. 6 where the number of reads per million (RPM) increases as the DNA fragments get longer. When the DNA length is factored in (Fig. 6B, reads per thousand bases per million (RPKM)), there should be a constant read level if DNA length did not affect library preparation or sequencing efficiency. For reads less than ~ 400 bp, there is a significant under-representation of DNA, probably due to purification methods favoring longer DNAs. As DNA gets longer, the reads increase in frequency above what is expected, though the variation by length is small. This increase could be due to DNA shearing that causes multiple fragments of longer DNAs to be present in the mix. However, the nature of adaptor addition makes prediction of the original length challenging if not previously known. Unlike the ligation-based method, the transposase-based method allows adaptors to be added randomly anywhere in the DNA so information on the original DNA length is lost. If DNAs are a defined composition, those DNAs can be assembled by combining reads. However, if the source of the DNA is unknown, there is no reliable way to determine the original length. The shorter DNA resulting from this method of adaptor addition can be seen in Fig. 1E and F.

**Figure 7:**
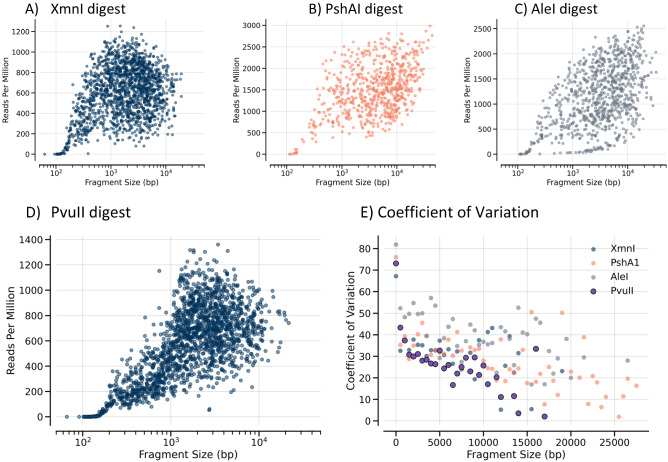
Read frequency versus length with Oxford Nanopore ligation-based library preparation. The normalized frequencies of all DNAs cut with XmnI (**A**), PshI (**B**), AleI (**C**), and PvuII (**D**) are shown as a function of length when the DNA is prepared using the ligation-based method for Oxford Nanopore. The coefficient of variation (**E**) for frequency for each digest as a function of length using 500 bp bins is also shown for each digest with the PvuII digest shown with larger symbols to distinguish more easily from the digests with variable end sequences.The normalized frequencies of all DNAs cut with XmnI (**A**), PshI (**B**), AleI (**C**), and PvuII (**D**) are shown as a function of length when the DNA is prepared using the ligation-based method for Oxford Nanopore. The coefficient of variation (**E**) for frequency for each digest as a function of length using 500 bp bins is also shown for each digest with the PvuII digest shown with larger symbols to distinguish more easily from the digests with variable end sequences.

**Figure 8:**
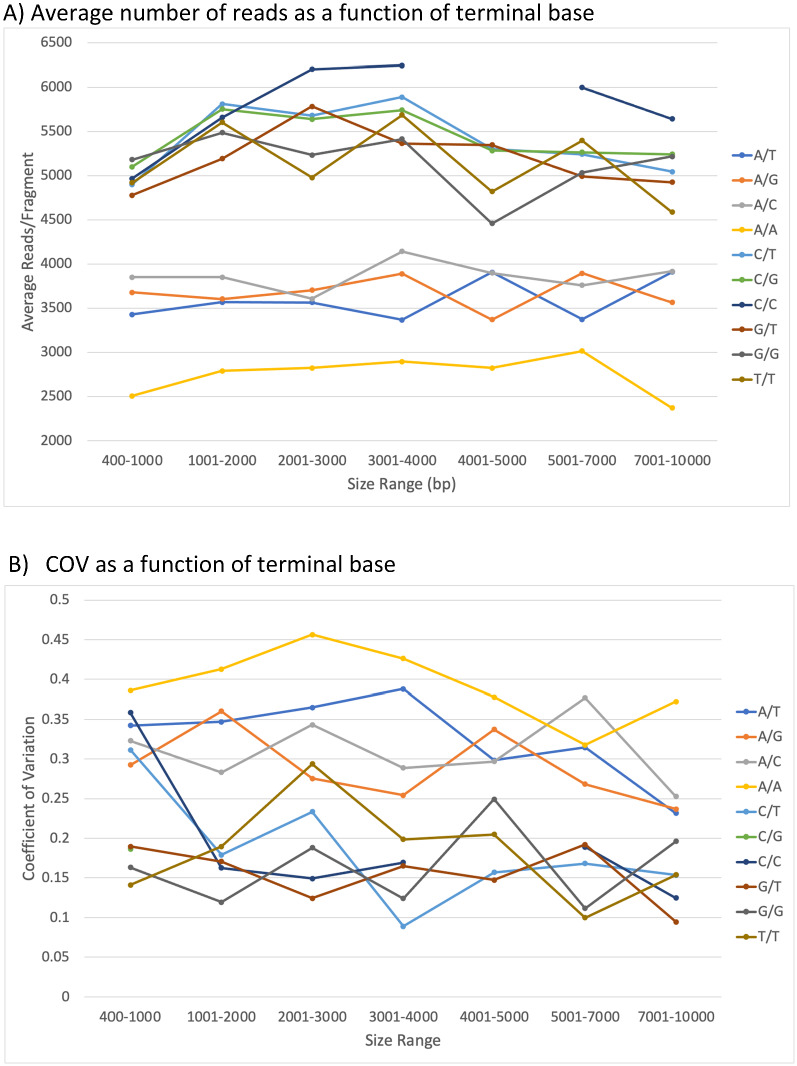
Read frequency as a function of length and 3’ terminal bases with Oxford Nanopore ligation-based library preparation. The average read count (**A**) and coefficient of variation (**B**) were calculated for all XmnI *E. coli* fragments from 400 to 10,000 bp. Length bins were selected to provide enough examples in each bin for all terminal base combinations. All points included > 5 fragments except for the 4001–5000 bp bin with CC termini which was omitted from the plots. All fragments with the same pair of 3’ ends were combined for analysis.

In addition to the transposase method, Oxford Nanopore also provides an alternative ligation-based method to attach sequencing adaptors, and, like the PacBio ligation methods, length information is preserved. As shown in Fig. 7, there is little apparent length dependence for read frequency above ~ 400 bp with the read frequency highly variable at any given length. Over a wide range of lengths, there is a greater than fourfold difference between the highest and lowest DNA read frequencies. Since three of the restriction enzymes used (AleI, PshAI, and XmnI) contain degenerate recognition sites that create DNA ends with different base compositions, it is possible that different ends could affect the efficiency during library preparation. There are 10 possible combinations of 3′ bases on each fragment. We analyzed XmnI fragments because most are less than 10 kb where random fragmentation is less of an issue. The average read count for fragments with each combination is shown in Fig. 8A for length bins ranging from 400 to 10,000 bp. DNAs with two 3′ As have uniformly the lowest frequency while DNAs with one 3′ A and another base form a group with slightly higher frequency. DNAs with no 3′ As form a third group with the highest frequency. This terminal base effect is independent of length. While somewhat noisier, the coefficient of variation displays an opposite effect with highest values for two 3′ As and lowest for no 3′ As (Fig. 8B). The effect is even more dramatic if the two terminal bases on each end are examined as shown in Supplemental Table 1 where pairs of 3′ AAs are six-fold less frequent than pairs of 3′ CCs. Supporting the impact of terminal bases, the coefficient of variation as a function of DNA length (Fig. 7) for the PvuII digest (uniform end sequence verified for > 95% of fragments by sequence) trends lower than that of the fragments with variable end sequences. This indicates that the terminal sequences may affect ligation or end processing, but there must also be other factors that impact sequencing efficiency.

The primary objective of the current work was to assess whether modelling of the distribution of sequencing efficiency (using number of counts as a proxy) based on initial fragment size was possible. Based on these results, we have focused efforts to generate quantitative estimates of DNA concentrations on the PacBio 2.1 protocol. The hundreds of *E. coli* DNA size markers we have used provide very high resolution for length calibration, but it would also require a large fraction of the available sequencing reads in many samples. A smaller reference like lambda would be more practical if it could provide similar accuracy. To address this question, we performed a PacBio sequencing experiment containing *E. coli* genomic DNA digested with PvuII restriction enzyme mixed with 1/10 (weight/weight) lambda phage genome digested with the same enzyme. Similar to what was observed in Fig. 1, the distribution of mean read size as function of the size of the reference fragment shows a pattern like *E. coli* with a maximum around 7400 bp mean read size. The distribution of read sizes (observed) and reference fragments (expected) compared to the expected theoretical distribution are shifted toward higher sizes as expected from the DNA purification. Despite the simultaneous sample handling, there are differences between equivalent sized lambda and *E. coli* fragments as shown in Fig. 2. The starting 48 kb lambda will be randomly sheared only rarely during sample preparation while the 4.6 Mb *E. coli* will be sheared throughout its length. This will impact the ability of the lambda DNA to predict the behavior of the same size *E. coli* DNA.

**Figure 9:**
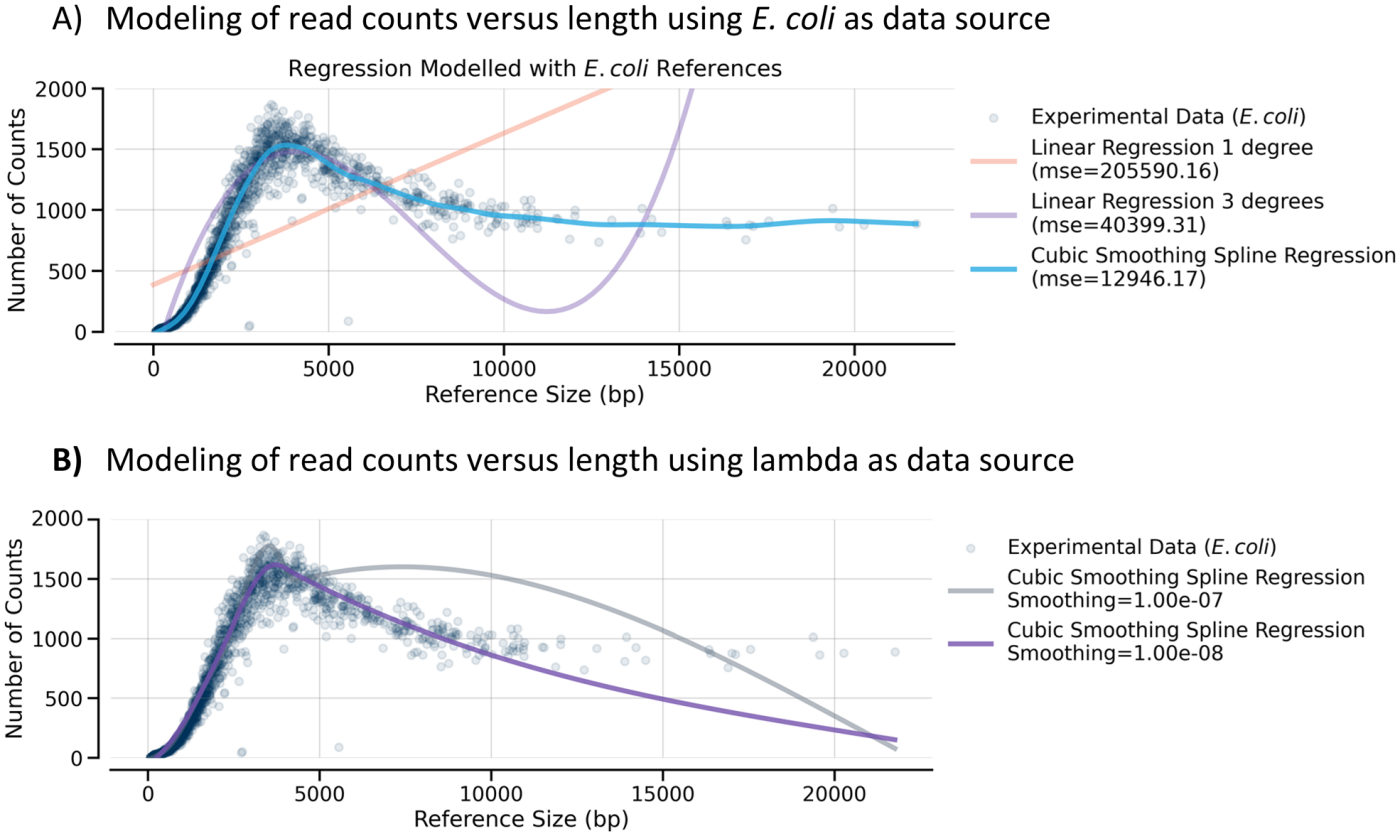
Fitting of DNA length versus read frequency. The read frequency of DNA fragments was fit using linear regression and cubic spline regression with *E. coli* DNA read counts as a data source (**A**). *E. coli* and lambda DNAs were mixed, cut with PvuII, and prepared using the 2.1 PacBio protocol. Because the linear regression methods did not model the *E. coli* DNA well, only cubic smoothing spline regression was used for lambda DNA. Lambda DNA was then used as the data source (**B**) and the spline regression generated with lambda data only was compared to the experimentally observed data for *E. coli* lengths and read counts. The darker blue line provides the best fit for both the modelled lambda and the experimental *E. coli* data.

In Fig. 9 (top panel), various regression methods for modeling the *E. coli* PvuII restriction digest fragment length data are shown. The lambda DNA data is used to generate mathematical models which are then compared to the experimental data from *E. coli* to assess how well the models fit real data. Due to the non-linear distribution of the data, linear regression methods fail to accurately model our results, as can be observed in the orange and purple lines in the top panel which attempt to model the experimental data using linear methods. Rather than linear regression, spline interpolation provides a better alternative, in which a group of low degree polynomials are used to fit subsets of the data. Cubic spline approximation (smoothing), a particular case of spline interpolation, seems particularly appropriate given the distribution of our data. When our *E. coli* restriction digestion fragment data is modeled with this method, we observe a good fit over the entire range of reference fragments (Fig. 9, top panel, blue line). Most importantly, the same type of regression, when applied to just the lambda phage genome restriction fragments, is also able to model satisfactorily the behavior of a wide range of *E. coli* restriction fragments despite the limited input of just lambda DNA for the model creation (Fig. 9, bottom panel). Overall, the data indicates that using a relatively low number of lambda phage fragments as a spike-in during PacBio Sequel II sequencing is sufficient for effective normalization models for the size range of interest, smaller than 7 Kbp. Because the read counts of DNA fragments larger than 10 kb is fairly constant, modeling in that range is not necessary. This methodology allows the detection of both sequence information and quantitative assessment of library diversity. We thus propose that the use of lambda fragment spike-ins alone and spline interpolation modelling should be considered whenever quantitative information is to be derived from PacBio Sequel II sequencing runs for fragment sizes less than 10 kb.

## Discussion

PacBio and Oxford Nanopore technologies have generally been applied to and optimized for situations that require long reads. Initially, technology limitations favored sequencing applications that did not require high read counts or high-fidelity base-calling. Short-read methods have often provided better base-calling accuracy and orders of magnitude more reads per unit cost, so they have served as the workhorse for applications like RNA-seq, ChIP-seq, and other quantitative methods. Long-read NGS has been more routinely employed in applications that cannot be easily carried out with short reads like structural variation detection, repetitive region assembly, and RNA isoform elucidation. These methods do not require the same high level of quantitation as applications like RNA-seq where millions of reads per sample are typically sought. When PacBio has been used in RNA-seq, potential issues with a length dependence of counting are usually addressed by comparing RNAs as ratios between samples thus cancelling out length as a quantitative factor, or by concatenating different RNAs so that no short reads occur^16,17^.

As a result of the low read counts and potential work arounds, not as much effort has been expended on characterizing factors that impact the quantitative accuracy of applications that require long reads. However, some long-read applications require an accurate estimation of molecular counts. One such application, assessing AAV vector quality, requires analysis of DNA lengths of ~ 200 bp to ~ 10 kb^18^ so short-read NGS is not practical. Quality control for viral gene therapy vectors is a key imperative and one that continues to evolve as new technologies are applied to the task. High quality DNA sequence information is important, both for confirming the sequence integrity of the vectors as well as for identifying potential DNA contaminants. rAAV is particularly challenging to deal with because it is both single-stranded (and different capsids contain different strands) and contains very GC-rich Inverted Terminal Repeats (ITRs) that are difficult to sequence and analyze using standard techniques^19^. AAV was first studied via single-molecule sequencing using Helicos^20^, but that technology generated short reads and could not span the whole genome. AAV can now be sequenced end-to-end using PacBio or Oxford Nanopore to verify its composition^11–14^. For analysis of rAAV as a human therapeutic, it is also critical to know whether other DNAs are present and to what degree. Thus, understanding how potential DNA contaminants behave when AAV is sequenced is important.

Because long-read technologies are slanted toward generating the longest reads possible rather than a complete catalog of what is present, each technology and, indeed, different versions of the same technology will yield very different patterns for a given DNA sample depending on the specific conditions used. For example, the normalization corrections we have observed using the PacBio Sequel II instrument are different than those observed previously with lambda DNA using the PacBio RSII instrument and older versions of reagents^12^. The RSII is known to have a very different length dependence for sequencing relative to the Sequel systems so this difference was expected^21^. Thus, calibrating library preparation methods needs to be an ongoing process to ensure that any change in behavior of relevant DNAs caused by technology or reagents can be accounted for when characterizing samples.

The avoidance of amplification with single-molecule sequencing provides the potential for accurate quantitation in diverse samples^22^. Sensitive quantitation is evidenced here by the ability to easily detect small variations in genome copy number in exponential versus stationary phase *E. coli*. NGS has been used to characterize origins of replication previously^23–25^ though often using sorted or synchronized cells and using short-read, amplification-based sequencing. Short reads bring the benefit of higher read counts though at the expense of amplification biases. Similarly, previous methods generally used sheared DNA, which provides more homogeneously sized DNAs while the restriction fragments examined here allow direct comparison of the same fragments. The > 50% increase in genomic copy number near the origin of replication versus the termination of replication that we observe is clear. There are additional patterns of copy number variation superimposed on the overriding effect of the origin/termination. The more localized patterns have not been examined further to determine whether there is structural or replication information that can be extracted from the short-range data.

We have used two different standard methods each for PacBio and Oxford Nanopore to understand whether a single sequencing reaction can be employed for identification and quantitation of AAV and contaminants. Full-length AAV is significantly shorter than the reads easily generated by both long-read technologies, and we have focused on just the AAV-relevant size range. Use of control DNAs to mimic the behavior of AAV and its contaminants must be done cautiously as evidenced by the differing behavior of lambda and *E. coli* DNAs. Even with the same size DNA, the much longer starting lengths of the *E. coli* genome led to a higher degree of random shearing than found with lambda or would be found with AAV.

Sequencing of AAV presents special challenges relative to most other DNAs. Individual DNA molecules emerge from the capsid as single-stranded DNA that ssDNA can hybridize with a second AAV molecule of opposite strandedness and potentially different length or sequence, making rigorous analysis challenging. The ITRs present on both ends could also lead to ligation or polymerase extension artifacts that are exacerbated if amplification is necessary. The base-specific ligation efficiency of the Oxford Nanopore approach would not be a significant issue for randomly sheared DNA but, if contaminants are a specific sequence, the DNA might appear to be over- or under- represented to a significant degree depending on the terminal sequences. Using the PacBio method that retains short DNAs provides full length sequences with predictable quantitation over the length range important for assessing both AAV and potential contaminants. Because the sequencing adaptors are attached via ligation to the DNA ends, it is possible to generate full-length sequences for both viral and contaminant DNA. The other methods tested provide less optimal behavior, although all provide useful information. Thus, as long as reliable calibration is carried out, these methods can be used to assess contamination of AAV and other gene therapy vectors. Ongoing monitoring of the behavior of different length DNAs used as calibration spikes will enable consistency across time and technology. With the most suitable PacBio 2.1 protocol, lambda spike-ins are sufficient for accurate quantitation so the complexities of using the much larger *E. coli* genome can be avoided.

## Supplementary Information

Supplementary Information.

## Data Availability

All sequence data has been deposited at SRA with submission ID SUB11557082.
