# Supplementary material for: DNA read count calibration for single-molecule, long-read sequencing
Source: Sci Rep. 2022 Nov 1;12:17257. doi: 10.1038/s41598-022-21606-5 (PMC9626564; doi:10.1038/s41598-022-21606-5)
Supplement: Supplementary file 1 — Supplementary Information. [file 41598_2022_21606_MOESM1_ESM.docx]

**Supplemental Table 1** Relative sequencing efficiency of ligation-based Oxford Nanopore libraries

In analyzing the terminal base impact, very short DNAs (<400 bp) are omitted because of their low read count due to their preferential removal by DNA purification. Very long DNAs (>10,000 bp) are also not used because of the higher likelihood that shearing could introduce random ends and eliminate any effect that depends on the initial ends.

With the two degenerate positions at both ends of each DNA, there are 136 different combinations of bases possible when both ends are included. These are listed in the table below (Seq) along the mean number of reads for those sequences (Mean Reads), the number of different DNAs falling into that category (N), and the ratio of read frequency relative to the sequence AA/AA (Ratio). The most detrimental bases are clearly 3’ AAs. When all XmnI fragments in the 400-10,000 bp range are grouped by terminal bases, the 17 DNAs with AA at both 3’ ends have the lowest mean read frequency. 15 of the lowest 16 combinations of bases include a 3’ AA on at least one end. At the high end of the frequency spectrum, the highest 15 base combinations all include at least one 3’ C and the highest has two 3’ CCs. The DNAs with 3’ CCs at both ends have >6x the mean frequency of the lowest combination with 3’ AAs at both ends.

| **Seq** | **Mean Reads** | **N** | **Ratio** | **Seq** | **Mean Reads** | **N** | **Ratio** | **Seq** | **Mean Reads** | **N** | **Ratio** |
| --- | --- | --- | --- | --- | --- | --- | --- | --- | --- | --- | --- |
| AA AA | 1228 | 17 | 1.00 | GT GT | 4259 | 4 | 3.47 | AG AT | 5295 | 13 | 4.31 |
| AA GA | 1818 | 17 | 1.48 | CA TG | 4311 | 9 | 3.51 | CG GG | 5312 | 11 | 4.33 |
| AA GT | 2044 | 18 | 1.66 | AG GC | 4390 | 9 | 3.57 | CG CG | 5323 | 4 | 4.33 |
| AA CA | 2124 | 19 | 1.73 | AG GG | 4407 | 5 | 3.59 | AG TT | 5357 | 12 | 4.36 |
| GA GA | 2174 | 6 | 1.77 | AT AT | 4430 | 6 | 3.61 | AT CG | 5414 | 11 | 4.41 |
| AA AG | 2192 | 13 | 1.78 | CG GC | 4434 | 5 | 3.61 | AT CT | 5414 | 17 | 4.41 |
| AA TT | 2448 | 21 | 1.99 | GC GG | 4479 | 10 | 3.65 | AC GG | 5420 | 11 | 4.41 |
| AA CG | 2455 | 14 | 2.00 | AG CA | 4491 | 8 | 3.66 | AT TT | 5441 | 8 | 4.43 |
| AA TC | 2465 | 15 | 2.01 | GG TA | 4500 | 18 | 3.66 | GT TC | 5464 | 5 | 4.45 |
| AA GC | 2477 | 18 | 2.02 | TA TT | 4500 | 17 | 3.66 | AC TA | 5497 | 11 | 4.48 |
| AA AT | 2495 | 27 | 2.03 | CA CG | 4541 | 10 | 3.70 | CT TT | 5503 | 6 | 4.48 |
| AA TG | 2513 | 19 | 2.05 | GT TG | 4542 | 12 | 3.70 | GG TC | 5547 | 7 | 4.52 |
| AA GG | 2530 | 14 | 2.06 | AG GT | 4545 | 10 | 3.70 | CT CT | 5570 | 2 | 4.54 |
| AA TA | 2581 | 15 | 2.10 | AT CA | 4545 | 14 | 3.70 | TC TT | 5596 | 5 | 4.56 |
| AA CC | 2729 | 8 | 2.22 | TA TG | 4581 | 13 | 3.73 | CT GG | 5622 | 7 | 4.58 |
| AA CT | 2800 | 7 | 2.28 | CT GC | 4657 | 8 | 3.79 | TT TT | 5626 | 6 | 4.58 |
| CA GA | 2852 | 13 | 2.32 | GC TG | 4678 | 8 | 3.81 | CC TG | 5630 | 7 | 4.58 |
| AA AC | 2939 | 17 | 2.39 | CG TA | 4722 | 18 | 3.85 | AG CG | 5665 | 12 | 4.61 |
| GA TA | 3092 | 20 | 2.52 | TA TC | 4755 | 14 | 3.87 | CC GC | 5672 | 2 | 4.62 |
| GA GT | 3160 | 11 | 2.57 | AT GC | 4756 | 11 | 3.87 | CG TC | 5678 | 16 | 4.62 |
| GA GC | 3295 | 11 | 2.68 | CT GT | 4768 | 6 | 3.88 | CG TG | 5722 | 11 | 4.66 |
| AG GA | 3318 | 8 | 2.70 | CC TA | 4781 | 10 | 3.89 | AT TC | 5756 | 14 | 4.69 |
| GA TT | 3372 | 10 | 2.75 | AT GG | 4788 | 6 | 3.90 | CG TT | 5757 | 10 | 4.69 |
| GA GG | 3400 | 11 | 2.77 | CA TC | 4790 | 12 | 3.90 | AG TC | 5785 | 10 | 4.71 |
| AT GA | 3543 | 19 | 2.89 | CT TA | 4799 | 12 | 3.91 | TC TG | 5853 | 9 | 4.77 |
| GA TG | 3636 | 14 | 2.96 | GC TC | 4806 | 7 | 3.91 | CG CT | 5867 | 8 | 4.78 |
| CA GT | 3665 | 7 | 2.98 | GG TG | 4817 | 6 | 3.92 | AC AG | 5868 | 12 | 4.78 |
| CT GA | 3705 | 12 | 3.02 | GG GT | 4818 | 7 | 3.92 | AG CC | 5931 | 2 | 4.83 |
| CC GA | 3773 | 7 | 3.07 | GC GC | 4902 | 5 | 3.99 | CC CT | 5949 | 3 | 4.84 |
| CG GA | 3809 | 19 | 3.10 | GC TT | 4931 | 14 | 4.02 | CT TG | 6021 | 6 | 4.90 |
| CA GC | 3845 | 11 | 3.13 | AC GT | 4940 | 7 | 4.02 | AC TG | 6063 | 10 | 4.94 |
| GC TA | 3949 | 13 | 3.22 | CC GG | 4951 | 5 | 4.03 | CT TC | 6072 | 5 | 4.94 |
| AC GA | 3950 | 21 | 3.22 | CA CT | 4954 | 12 | 4.03 | AT CC | 6157 | 11 | 5.01 |
| TA TA | 3965 | 15 | 3.23 | AC GC | 4960 | 10 | 4.04 | CC CG | 6194 | 6 | 5.04 |
| GT TA | 3971 | 16 | 3.23 | GT TT | 4973 | 8 | 4.05 | TC TC | 6283 | 5 | 5.12 |
| AT TA | 3989 | 13 | 3.25 | AC CA | 5004 | 12 | 4.07 | AC AT | 6289 | 14 | 5.12 |
| GA TC | 4048 | 10 | 3.30 | AG CT | 5014 | 8 | 4.08 | AC TC | 6311 | 5 | 5.14 |
| GC GT | 4093 | 5 | 3.33 | TG TG | 5047 | 8 | 4.11 | AC CC | 6335 | 10 | 5.16 |
| CA TT | 4100 | 11 | 3.34 | AG TG | 5172 | 17 | 4.21 | CC TT | 6337 | 5 | 5.16 |
| CA CA | 4124 | 11 | 3.36 | AT GT | 5173 | 7 | 4.21 | CC TC | 6338 | 5 | 5.16 |
| GG GG | 4124 | 2 | 3.36 | CC GT | 5187 | 14 | 4.22 | AC TT | 6362 | 7 | 5.18 |
| CA TA | 4174 | 11 | 3.40 | CA CC | 5197 | 6 | 4.23 | AC AC | 6420 | 4 | 5.23 |
| AG AG | 4205 | 1 | 3.42 | TG TT | 5221 | 10 | 4.25 | AC CT | 6446 | 4 | 5.25 |
| CG GT | 4217 | 7 | 3.43 | GG TT | 5256 | 7 | 4.28 | AC CG | 6517 | 7 | 5.31 |
| CA GG | 4239 | 11 | 3.45 | AT TG | 5266 | 14 | 4.29 | CC CC | 7390 | 3 | 6.02 |
| AG TA | 4248 | 13 | 3.46 |  |  |  |  |  |  |  |  |

**Supplemental Figure 1** Fragment Analyzer traces of different library DNAs


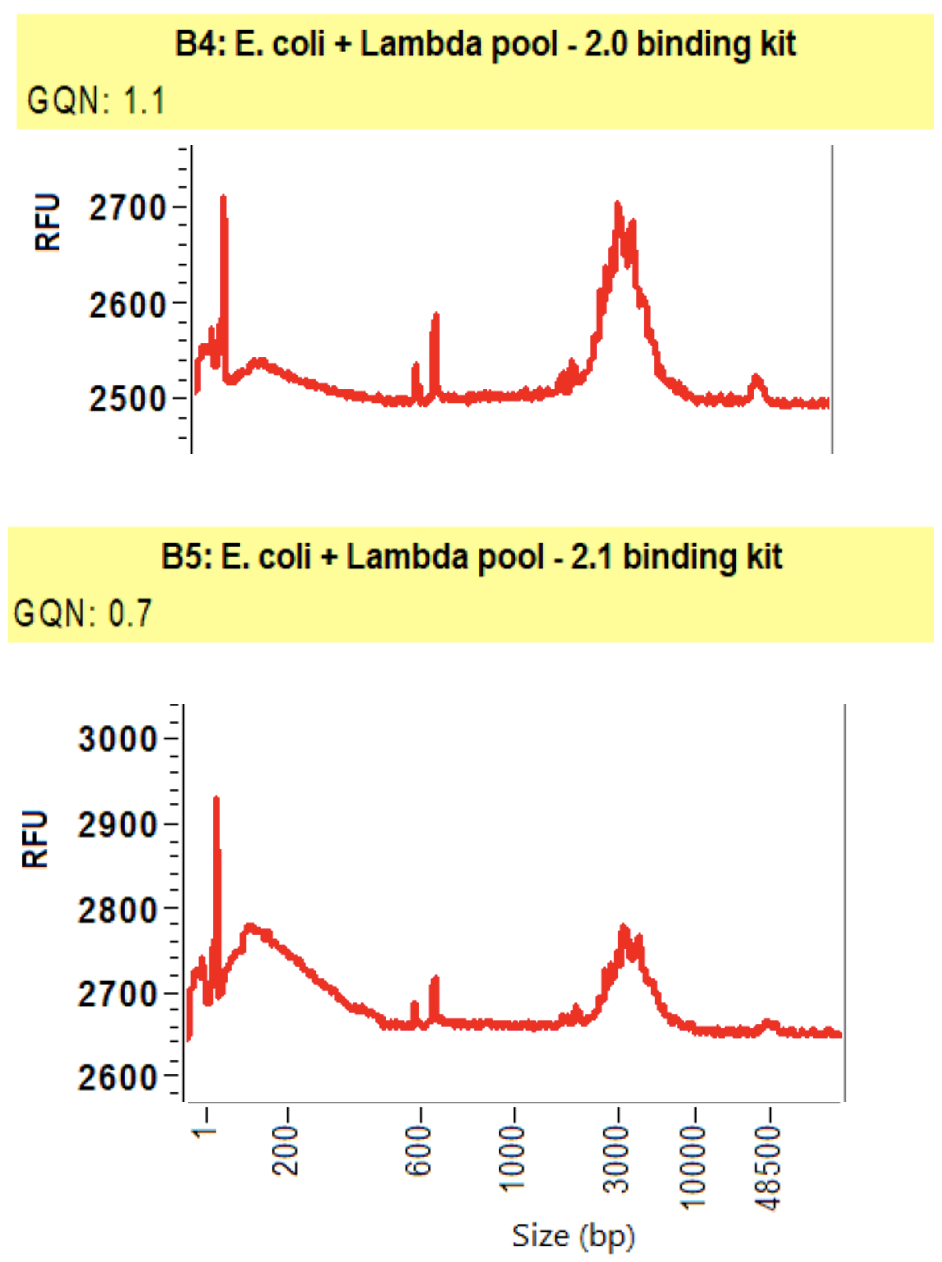


Pooled lambda/*E. coli* samples purified using the 2.0 (top) or the 2.1 (bottom) protocol were run on a Fragment Analyzer. Approximate lengths of the DNA fragments are shown below.

**Supplemental Figure 2**:

A) Lengths for selected XmnI-digested fragments using Oxford Nanopore ligation method


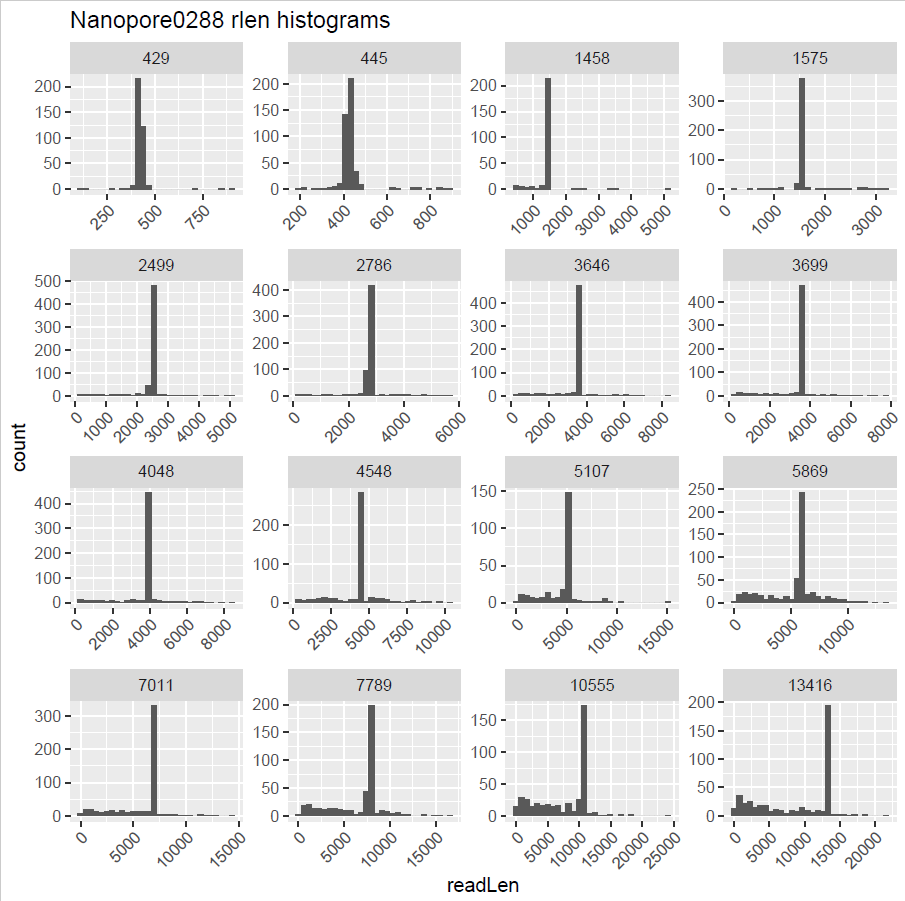


B) Lengths for selected XmnI-digested fragments using Oxford Nanopore transposase method


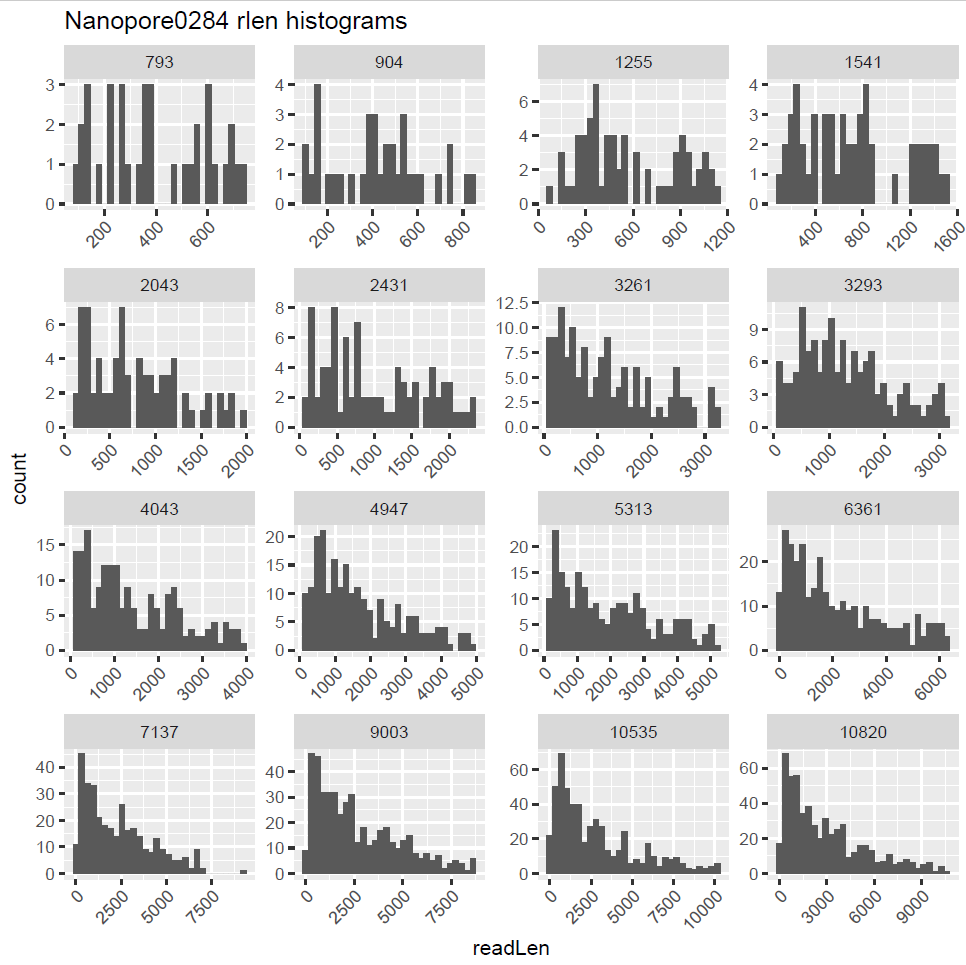


XmnI fragments of varying size sequenced using either the ligation method (A) or the transposase method (B) were counted for length. The vast majority using the ligation method were the expected length (shown above the length distribution for each fragment) while the transposase method generated a range of sizes less than full-length. The observed read length is shown on the horizontal axis while the relative number of each length is shown on the vertical axis.

**Supplemental Figure 3**: Fragment analyzer traces for uncut lambda DNA

**
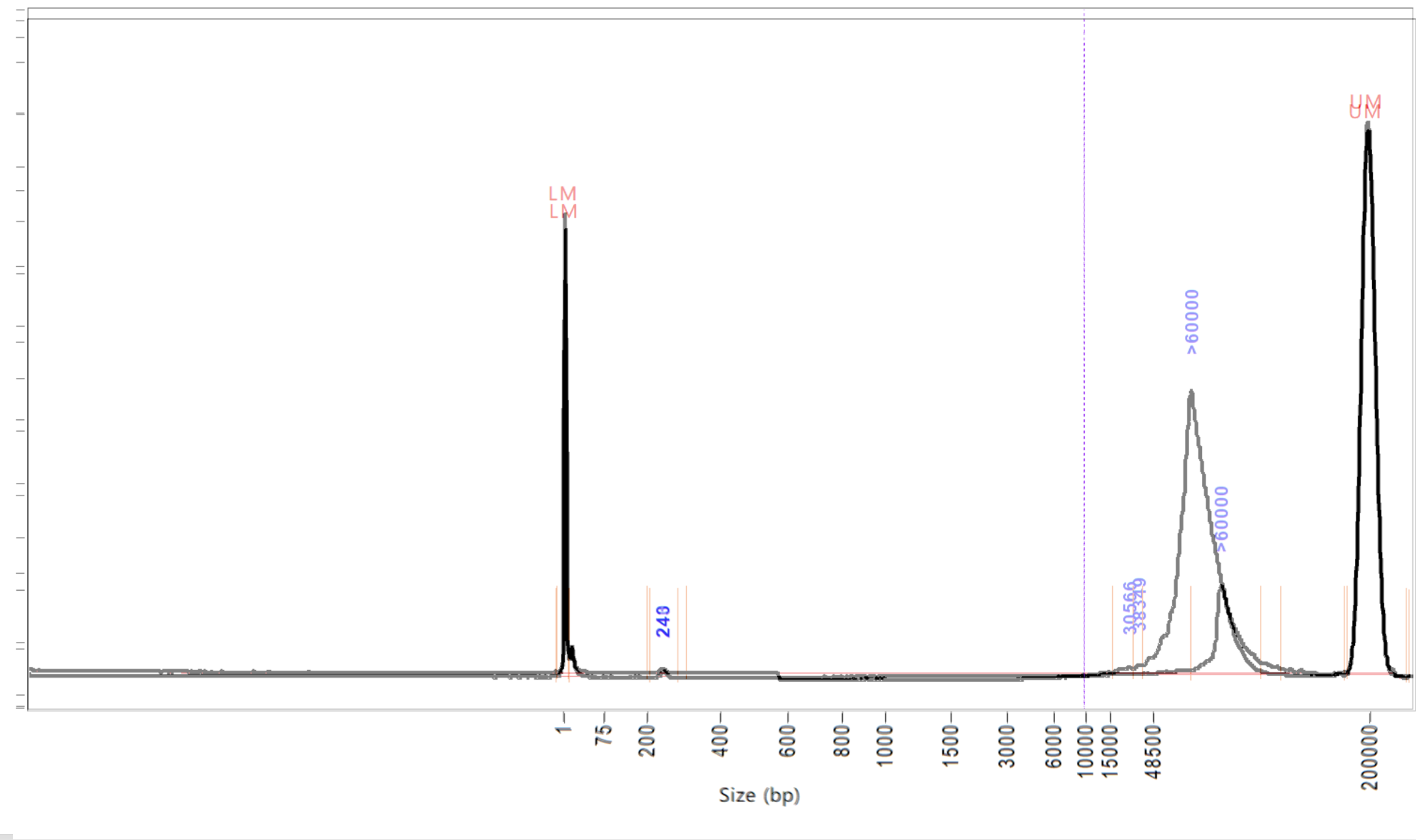
**

Uncut lambda DNA was treated with Taq polymerase and dNTPs in order to fill in the sticky overhangs at the cosN sites. 1 ng lambda each of untreated and filled in lambda was run on the Fragment Analyzer and the traces superimposed on each other based on the positions of the upper and lower markers. The run with lower amounts and longer DNA is the untreated sample. The larger size and apparently lower amounts of DNA indicate that most of the untreated DNA was too large to be detected in the Fragment analyzer and the larger size indicates significant concatenation via the cosN sites.

**Supplemental Figure 4** Weighted, rolling average ratio of exponential to stationary phase read frequency

Reads from XmnI and PvuII digests from fragments >100 bp with >20 reads were each normalized within the exponential and stationary phase DNA samples. The ratio of exponential/stationary phase reads for each fragment as a function of starting genomic position is shown. A weighted, rolling average is shown in A. For the weighted, rolling average, the central DNA is weighted as 1 with adjacent DNAs on either side weighted at 0.96, the next adjacent DNAs weighted at 0.92, etc. to include a total of 49 adjacent DNAs in the average. The starting genomic position of the central DNA fragment is plotted versus the ratio of exponential/stationary phase reads. The XmnI/PvuII results (B) are a combination of those fragments ordered by starting genomic position.

A)


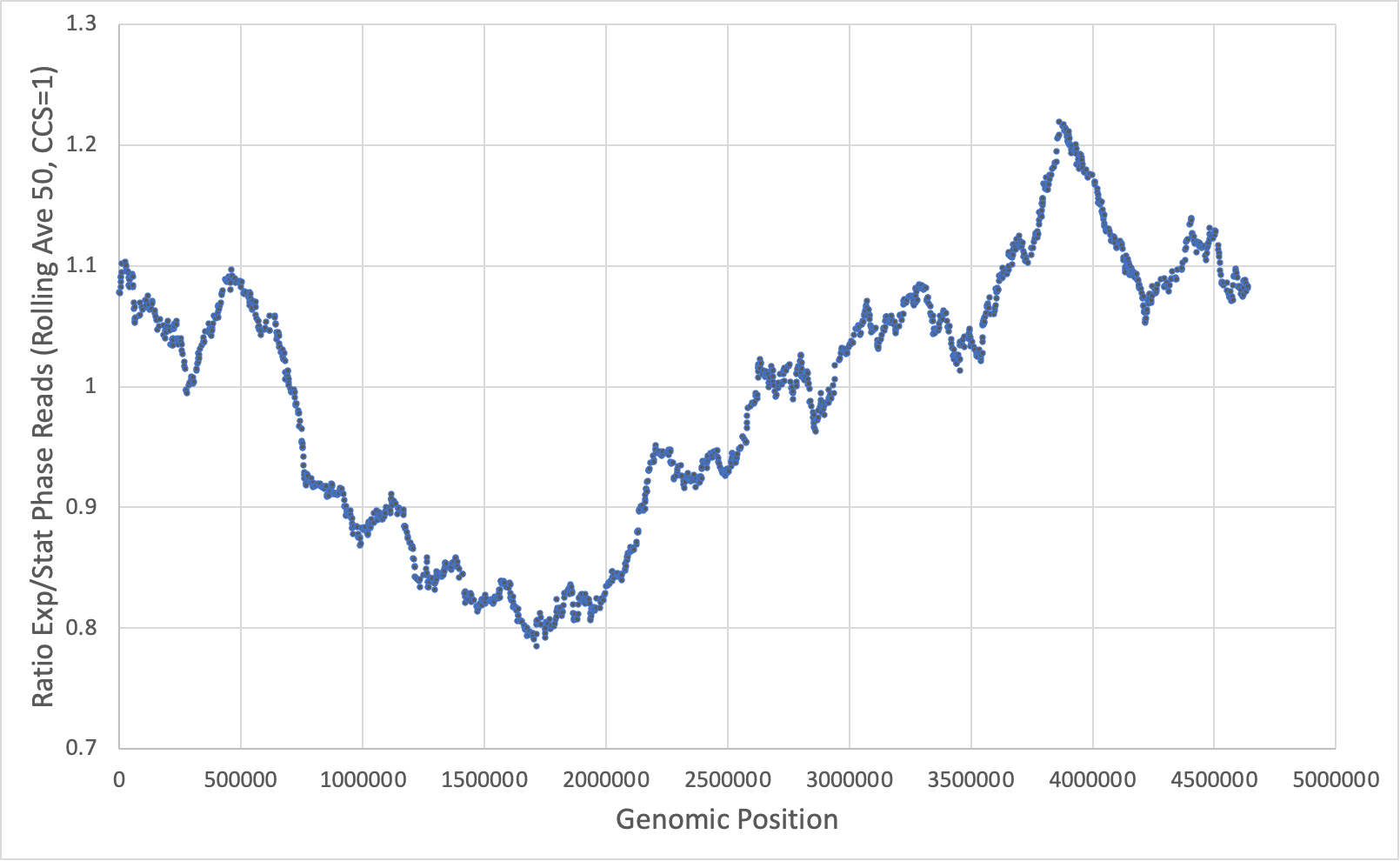


B)


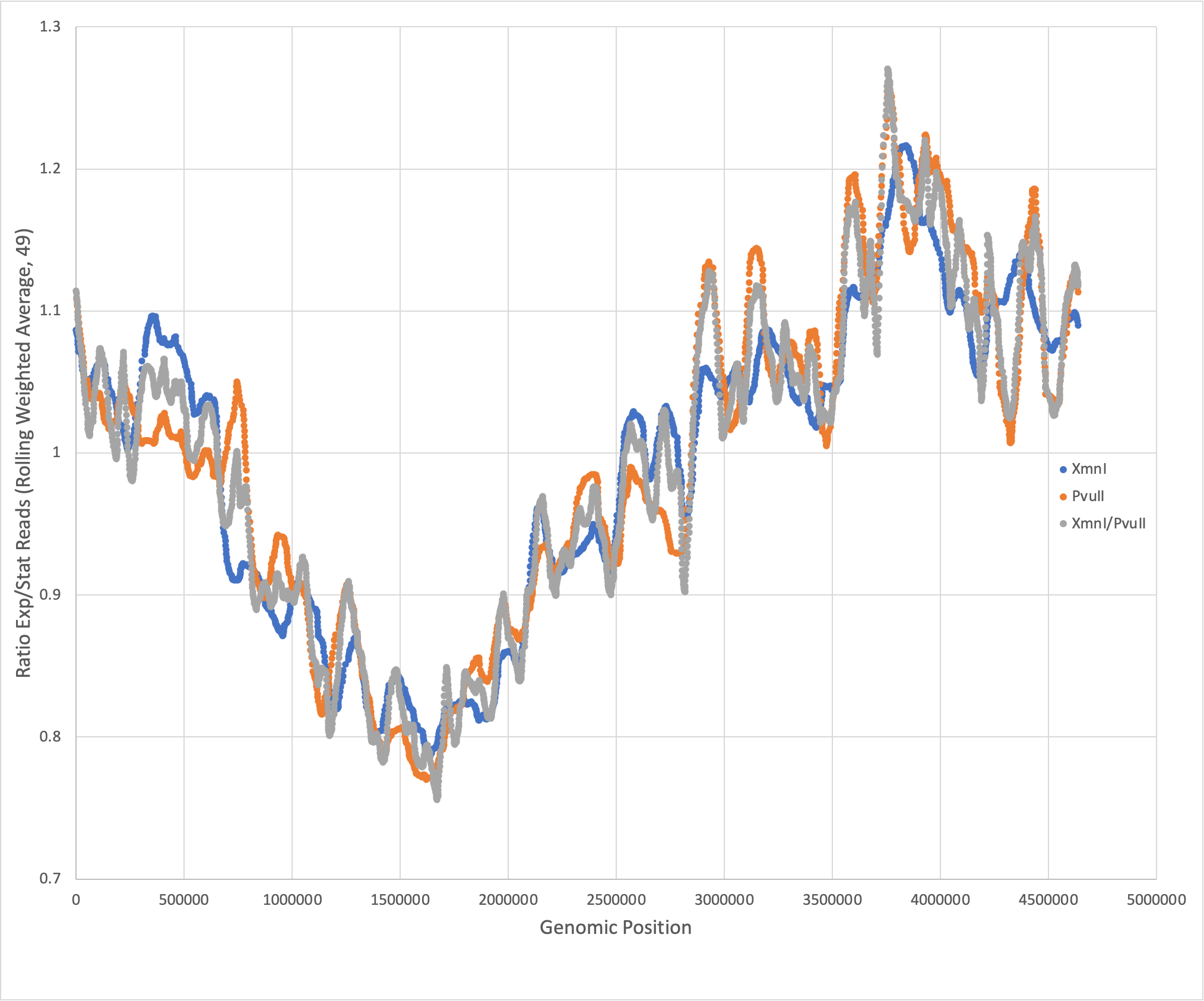


**Supplemental Figure 5:** Rolling, weighted average of DNA sizes


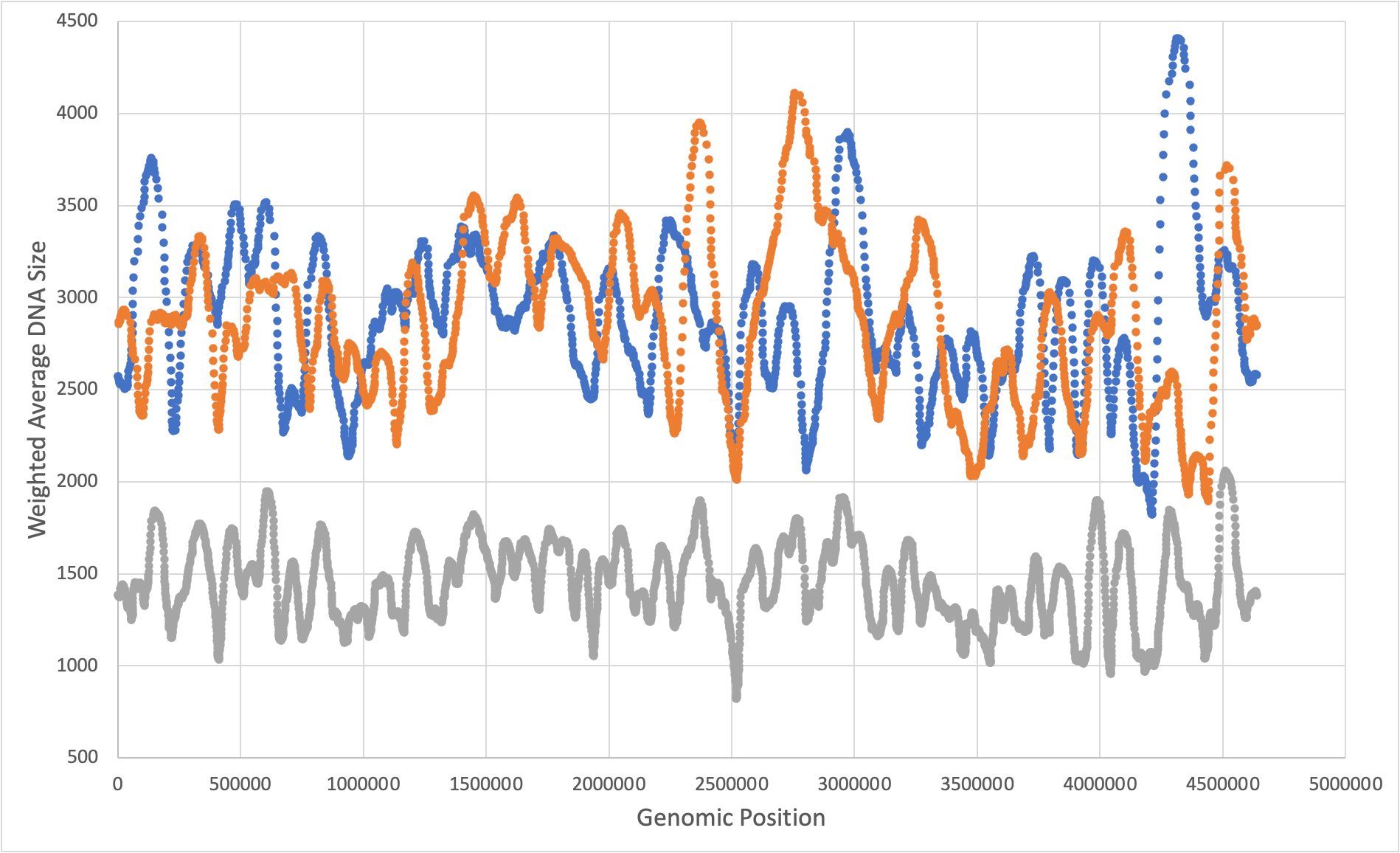


The same XmnI and PvuII fragments >100 bp with >20 reads used in Supplemental Figure 4 were analyzed. The weighted, rolling average of lengths for each fragment as a function of starting genomic position is shown. The central DNA is weighted as 1 with adjacent DNAs on either side weighted at 0.96, the next DNAs weighted at 0.92, etc. to include a total of 49 adjacent DNAs in the average. The relatively flat relationship suggests DNA length does not affect the results in Supplemental Figure 4.
